# Interferometric Backward Third Harmonic Generation Microscopy for Axial Imaging with Accuracy Beyond the Diffraction Limit

**DOI:** 10.1371/journal.pone.0094458

**Published:** 2014-04-07

**Authors:** Daaf Sandkuijl, Lukas Kontenis, Nuno M. Coelho, Christopher McCulloch, Virginijus Barzda

**Affiliations:** 1 Department of Physics and Institute for Optical Sciences, University of Toronto, Toronto, Ontario, Canada; 2 Department of Chemical and Physical Sciences, University of Toronto Mississauga, Mississauga, Ontario, Canada; 3 Matrix Dynamics Group, University of Toronto, Toronto, Ontario, Canada; University of New South Wales, Australia

## Abstract

A new nonlinear microscopy technique based on interference of backward-reflected third harmonic generation (I-THG) from multiple interfaces is presented. The technique is used to measure height variations or changes of a layer thickness with an accuracy of up to 5 nm. Height variations of a patterned glass surface and thickness variations of fibroblasts are visualized with the interferometric epi-THG microscope with an accuracy at least two orders of magnitude better than diffraction limit. The microscopy technique can be broadly applied for measuring distance variations between membranes or multilayer structures inside biological tissue and for surface height variation imaging.

## Introduction

Nonlinear microscopy is an invaluable tool in the study of biological samples. In particular, second harmonic generation microscopy is uniquely suited for imaging collagen, myosin fibrils, and starch granules [1], while third harmonic generation visualizes structural interfaces, lipid bodies and pigmented photosynthetic structures [2]. One of the main advantages of nonlinear microscopy is that the nonlinear signal generation is confined to the diffraction-limited focal volume, which leads to inherent optical sectioning and allows for 3D imaging of structures embedded in biological tissue. Furthermore, multiple nonlinear optical signals can be generated and detected at the same time, leading to multimodal harmonic generation and fluorescence microscopy [3].

One of these harmonic signals, third harmonic generation (THG), originates from inhomogeneities in the refractive index and/or third-order nonlinear susceptibility in isotropic biological materials. As a result, THG is commonly used as a probe for interfaces between different media [4], [5]. We recently showed numerically that backward-directed THG (epi-THG) from an interface is mostly caused by reflection at the boundary of the forward THG (F-THG) generated before the interface [6]. When multiple interfaces are present the reflected THG signals will interfere. Therefore, interference effects in epi-THG can be used to determine the spacing between interfaces with accuracy beyond the diffraction limit, and may be applied in the imaging of layered structures, or for surface topography measurements when the topography of one of the interfaces is known (e.g. a flat sample coverslip or an external surface). Similar interference effects in backward-detected signal were previously observed in coherent anti-Stokes Raman scattering microscopy (CARS), although in that case the signal was not due to reflections from interfaces but rather due to interference of direct backward CARS signals [7]. In addition, endoscopic applications of harmonic generation microscopy are becoming more popular [8], [9]. In endoscopes only epi-THG can be detected, and it is therefore important to understand epi-THG signals from layered structures.

In this work, we demonstrate experimentally for the first time that epi-THG can be used to measure surface height topography of a sample with 5 nm accuracy, far below the diffraction limit. We explore the application of interferometric THG (I-THG) microscopy for imaging biological samples by visualizing the thickness variation of fibroblasts attached to a fibronectin-coated microscope coverslip.

## Materials and Methods

### 1. Sample preparation

Three types of samples were prepared for the experiments. For the first type of samples, cleaned glass coverslips (22×22 mm, VWR) were coated with 10 μg/mL of human plasma fibronectin (Sigma) for 30 min at 37 °C and then washed with Dulbecco's modified Eagle medium (DMEM) for immediate use. Mouse NIH-3T3 cells, cultured at 37 °C in complete DMEM medium containing 10% fetal bovine serum and antibiotics, were detached from a confluent flask with trypsin/EDTA (Invitrogen). Residual trypsin activity was blocked with complete medium. Cells were seeded in 6 well tissue culture plates containing the fibronectin-coated glass coverslips for 12 h. At the end of incubation, the cells were fixed with 4% paraformaldehyde (20 min) and mounted on a microscope coverslip and covered with another coverslip.

**Figure 1:**
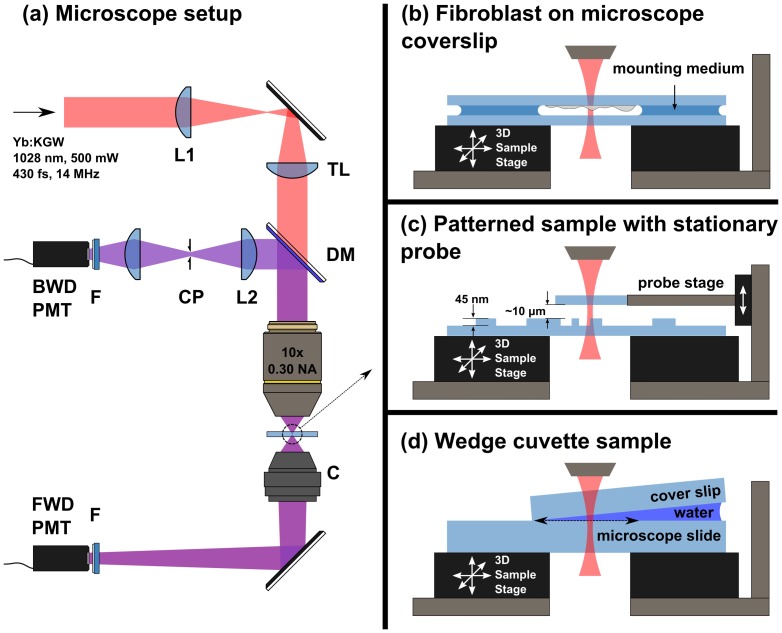
Overview of the microscope setup. L1, L2: lenses, TL: tube lens, DM: dichroic mirror, CP: 75 μm confocal pinhole, F: Band pass filter 340-10 nm, C: collection objective, BWD and FWD PMT: backward and forward photomultiplier tube detectors. (b) Overview of the fibroblast scanning geometry. The top and bottom surfaces of the fibroblast reflect THG, which causes interference in epi-THG. (c) Overview of the patterned sample scanning geometry. An external probe provides the second surface for reflection of THG. The probe is held at a fixed distance from the sample holder. (d) Overview of the wedge geometry. The arrow indicates the approximate scanning direction used to obtain the data in Fig. 3. (See text for details.)

The second sample was a glass microscope coverslip (VWR VistaVision) with patterns of specified depths etched into the surface (Fig. 1b). The patterns were deposited on the surface of the coverslips using electron beam nanolithography, and were subsequently etched with a buffered oxide etch (BOE) solution of 1∶10 concentration of 49% HF in water to 40% NH_4_F in water. In this work we used a pattern depth of 45 ± 5 nm, as measured with a profilometer (TencorAlphaStep 200).

The third sample was a wedge cuvette assembly, which consisted of a 175 μm-thick coverslip on top of a microscope slide, with one end of the coverslip raised by another coverslip and the gap filled with distilled water (Fig. 1c). Before filling the wedge with water, the wedge angle and spacing were measured by determining the glass air interface position with F-THG axial profile scans using a 0.75 NA excitation objective at several positions along the wedge. The wedge angle was determined to be 0.84 ± 0.03 degrees (95% conf. int.).

### 2. Microscope setup

The I-THG microscope is based on the nonlinear microscopy setup described in detail elsewhere [10], with modifications which are depicted in Fig. 1. Briefly, we used a home-built Yb:KGW laser operating at 1028 nm wavelength, with pulse duration of 430 fs and repetition rate of 14.3 MHz [10]. A 0.3 numerical aperture (NA) objective (Olympus UPlanFL N 10x) was used for excitation and epi-THG detection. The pulse energy at the focus of the objective was set to 5 nJ for all measurements, which should not affect cell viability at our laser wavelength and low focusing NA [11]. The epi-THG signal was focused through a 75 μm confocal pinhole by a 150 mm lens to ensure that no out-of-focus reflected or scattered F-THG was detected in the epi-THG detection channel. The F-THG signal was collected using a custom-built collection objective with a numerical aperture of 0.85, which was optimized for transmission of the THG wavelength. A three-axis piezoelectric stage (Physik Instrumente P-563.3CD) was used to provide sample scanning. Due to our use of sample scanning, the acquisition time for the images was on the order of ten seconds, which makes the microscopy technique applicable to live cell imaging. Beam scanning could be used with descanned detection for increased imaging throughput.

For I-THG microscopy two interfaces need to be present within the focal volume to create interference in the epi-THG signal. For many samples, such as the fibroblasts grown on a microscope slide, the two interfaces are intrinsic to the sample (*i.e.*, the top and bottom cell membrane of the fibroblast). For samples without both interfaces present, or in case a specific surface topology is to be imaged, a ‘probe’ interface with known surface structure can be introduced. Therefore, for measurements on I-THG from the patterned glass coverslip we introduced a second glass coverslip as a probe. The bottom interface of the probe was located close to the top interface of the sample (Fig. 1b). The distance between the probe and the sample holder was kept stable by a custom-designed mount which allows for translation and tilt alignment. In addition, a custom plane scanning algorithm was developed for the piezo-electric stage to scan the sample in a plane tilted with respect to the vertical axis, so that fringes could be induced or removed at will. As mentioned, for the experiments on the fibroblast sample and the wedge cuvette sample (see Fig. 1c) the probe was not required since both of the required interfaces were intrinsic to the sample.

## Results and Discussion

### 1. Principle of I-THG microscopy

The I-THG microscope relies on two interfaces being located in the focal volume of the excitation objective. Forward THG (F-THG) is generated in the first material encountered by the excitation beam. This F-THG is reflected by both interfaces (*e.g.*, the top and bottom cell surfaces). Interference between these two reflections leads to a setup similar to a Fabry-Pérot etalon. Therefore, the magnitude of the epi-THG signal depends on the distance between the interfaces with a fringe period on the order of half the THG wavelength, or one-sixth of the excitation wavelength, implying structures much smaller than the excitation wavelength can be imaged [6].

### 2. Fibroblast sample

**Figure 2:**
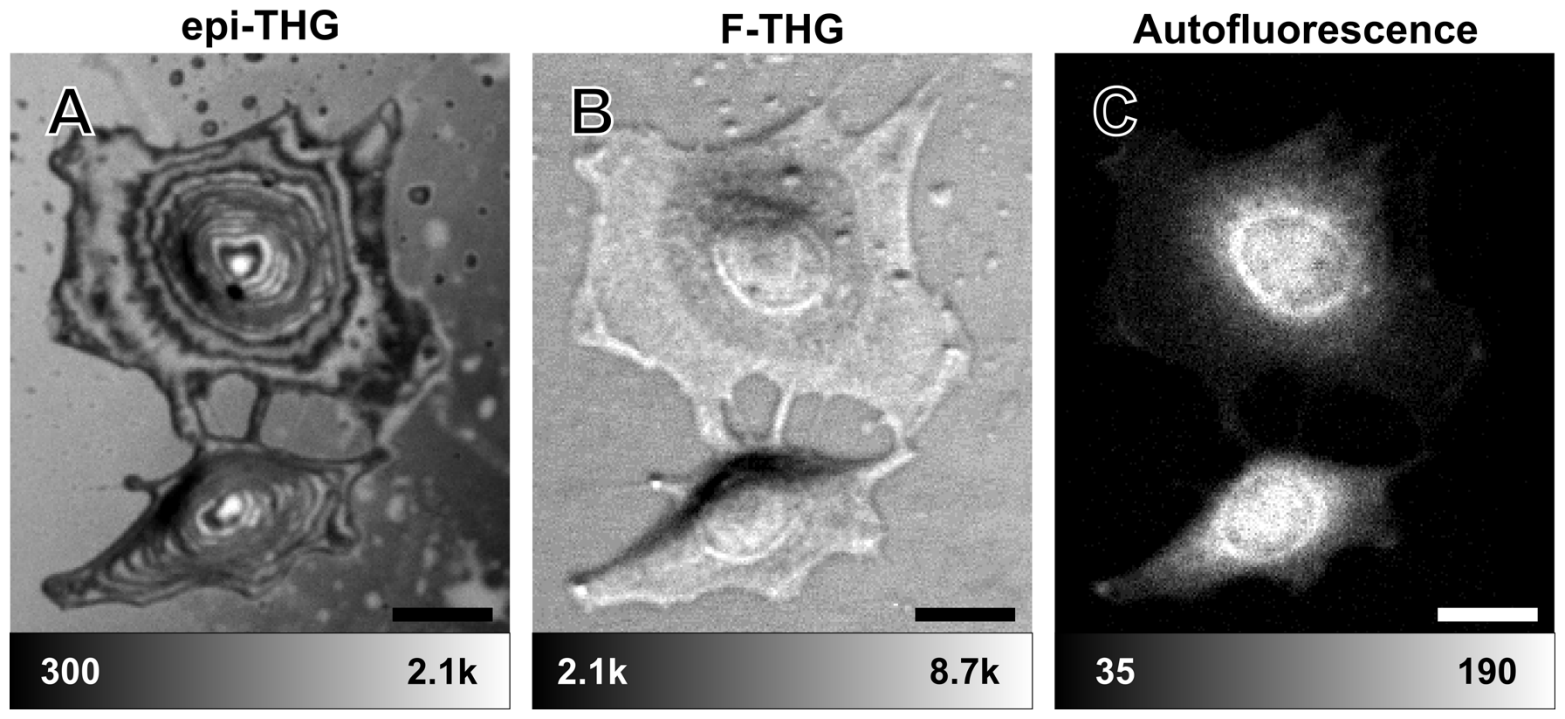
Epi-THG, F-THG and autofluorescence from unstained fibroblasts on a fibronectin-coated coverslip. Fringes in epi-THG (a) are clearly visible, with each fringe representing a height difference of approximately 190 nm. The F-THG (b) contains different information from the epi-THG image. Autofluorescence (c) was collected between 525 and 630 nm. The scale bar is 20 μm. Intensity scales show photon counts. The pixel dwell time is 6 ms.

The application of I-THG microscopy for biological samples was explored by imaging unstained fibroblasts on a fibronectin-coated microscope coverslip. Since the fibroblasts adhered to the surface of the coverslip, the interface between the glass and the cell membranes can be used to generate F-THG. Interference between reflections from the glass and cell membrane interfaces introduces fringes in the epi-THG signal, and since the glass surface is flat the fringes indicate differences in the cell height profile. Note that the top cell interface and the glass interface have the highest refractive index contrast and therefore dominate the contribution to the epi-THG interference pattern. The bottom cell interface (the interface closest to the fibronectin-coated cover slip) has a much lower refractive index contrast and thus will only produce a weak reflection. Furthermore, the cell contains internal organelles containing interfaces, and these are expected to reflect the THG signal as well. However, we expect the signal due to these reflections to be much weaker than that of the two interfaces mentioned above for several reasons. These internal interfaces are often curved with radii of curvature of the same order of magnitude or smaller than the wavelength of light, meaning that epi-THG reflected or scattered from these interfaces will defocus rapidly and only small areas with membranes perpendicular to the excitation beam propagation direction will contribute to the observed interference pattern from the specimen. In addition, the refractive index contrast of these interfaces is often lower than for the glass-specimen and upper cell membrane interfaces, and they often are part of a layer structure with thickness much smaller than the wavelength of light, leading to destructive interference in the reflected THG. Such internal interfaces could however be probed by modifying the experimental conditions, such as by removing one of the interfaces considered or by changing the focusing conditions of the excitation beam. Fig. 2a shows an epi-THG image of a fibroblast cell, with fringes indicating surface height differences of approximately 190 nm. Closed fringes denote equal heights, and thus the image can be treated as a topographic cell thickness map. The arrangement of the closed fringes outlines the overall shape of the cell: the top of the cell bulge corresponds to the location where the fringes converge, and the increase in fringe spacing at the filopodia denotes a change in surface slope of the stretched cell periphery. While the fringe pattern shown in Fig. 2a alone is not sufficient to construct an unambiguous height map, quantitative height maps can be generated in several ways: for instance, multi-step phase reconstruction [12], fringe shifting by sample tilting, or simultaneous multiple-wavelength excitation can be used. The error on such a height measurement can be estimated from the visibility of the fringes in Fig. 2a and from the possible refractive index uncertainty. For the peripheral regions the fringes are well resolved and refractive index uncertainty has limited influence due to the short optical path length between reflections, and we estimate the height measurement error to be at most a quarter of a fringe spacing, or less than 50 nm. For the nuclear regions the fringes are less well resolved and the optical path length between reflections is longer, leading to an estimate of the height measurement error of less than two fringe spacings.

Due to the interferometric nature of the technique, the signal relies on the relatively parallel alignment of the top cell surface with the glass surface, which causes steep parts of the cell to appear dark due to insufficient overlap between the back-reflected THG signals from the interfaces. On the other hand, F-THG is observed from the entire cell, with lower signal at the nucleus. However, it is clear that epi- and F-THG contain different information, and could be used as complementary techniques. In addition, Fig. 2c shows simultaneously detected autofluorescence from the cells, collected between 525 and 630 nm using a band-pass filter on a separate photomultiplier tube. This demonstrates that fluorescence staining and other nonlinear contrast mechanisms such as second harmonic generation (SHG) can be implemented simultaneously, providing more information regarding the sample.

The epi-THG imaging technique can be used for samples where slowly varying interface positions have to be observed with high accuracy. Examples include imaging of the topography of the cell plasma membrane, and cell walls in plant cells as well as measurement of the topography of cuticular wax layers on the leaf surface. In addition, combining the epi-THG technique demonstrated here with polarization-resolved SHG imaging [13] would enable new research in cell mobility of fibroblasts on collagen structures.

### 3. Patterned glass samples

**Figure 3:**
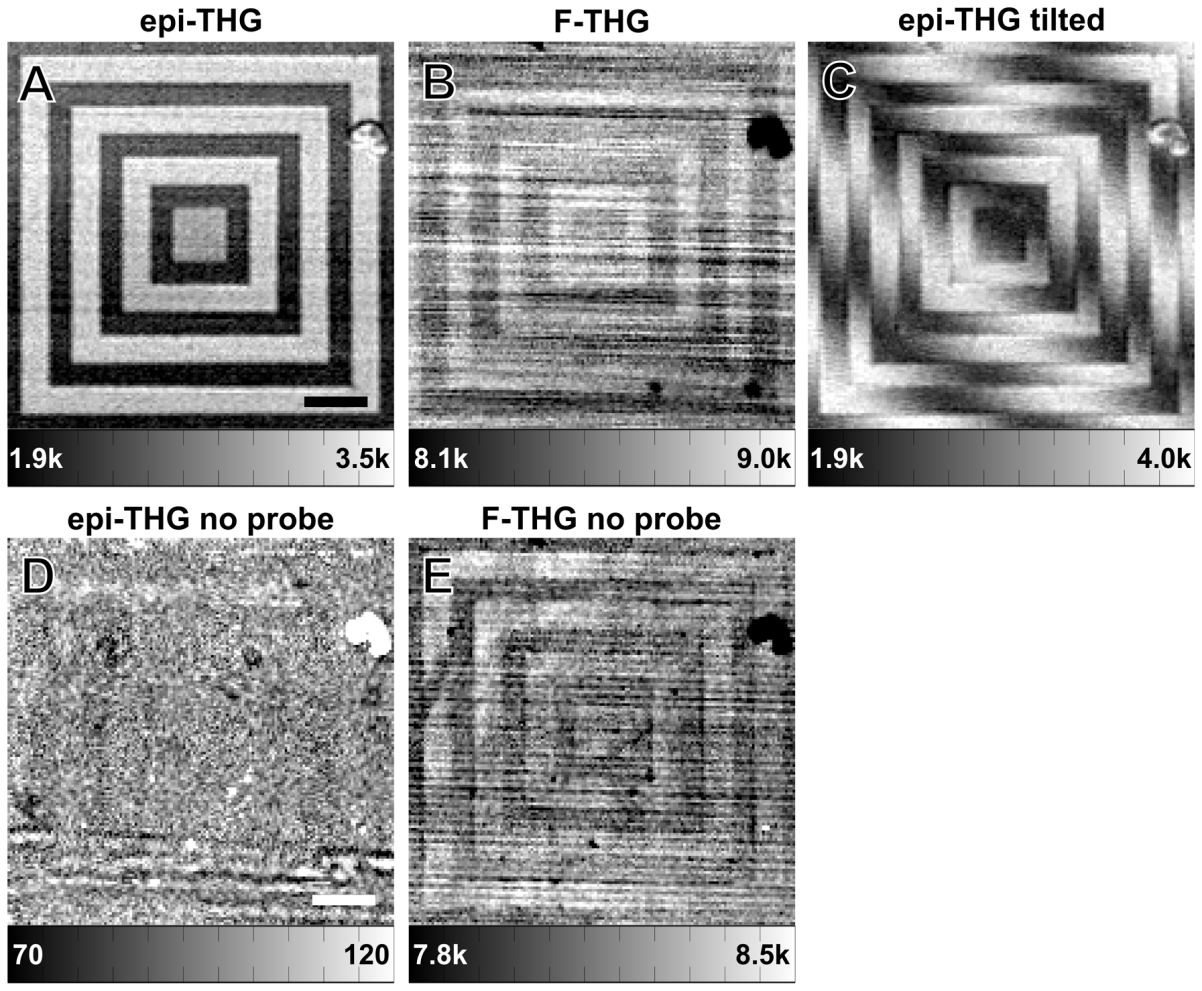
Epi-THG and F-THG signals from a 45-nm deep pattern etched in glass. (a) Epi-THG and (b) F-THG images from a patterned glass coverslip, imaged without tilt. (c) Epi-THG image from the patterned glass sample, with the scanning plane tilted by 0.13° with respect to the excitation direction. The pattern depth was determined to be 49 ± 3 nm, in good agreement with the depth of 45 ± 5 nm as measured with a profilometer. (d) Epi-THG and (e) F-THG images from the patterned glass sample with no probe present. In this case the pattern is not visible in epi-THG. Note that streaks in the F-THG images are scanning artifacts visually magnified by the dynamic range of the closely spaced values of the color bar. The scale bars represent 25 μm. The pixel dwell time was 30 ms. Color scales show photon counts.

In order to better demonstrate the features of the interferometric epi-THG microscopy a patterned glass sample was imaged with the I-THG microscope (Fig. 3). A ‘probe’ surface was positioned near the top surface of the sample (see Fig. 1b and Materials and Methods). The etched pattern, which was measured with a profilometer to be 45 ± 5 nm deep, is clearly distinguishable in the epi-THG image (Fig. 3a), with a large contrast-to-noise ratio of 11.3. The pattern is also visible in the F-THG image (Fig. 3b). This is expected from the Fabry-Pérot effect of the two glass/air interfaces, which modulates the transmission of fundamental light intensity as a function of the distance between the interfaces. However, the contrast-to-noise ratio is far worse than for the epi-THG images (0.7 vs. 11.3), since the modulation depth of the transmission of the excitation beam intensity by the two interfaces is much smaller than the modulation of the reflected THG by the interfaces. Without the probe interface present the pattern is barely visible in F-THG and practically indistinguishable in epi-THG (Figs. 3d and 3e). The extinction ratio is approximately 2, which indicates that the back-reflected THG from both interfaces overlaps well, and that other interfaces outside of the focal volume do not contribute significantly to the I-THG signal. This is expected from the fact that THG is only generated in the focal volume due to the nonlinear signal dependence, also known as inherent optical sectioning, and due to the presence of the confocal pinhole.

**Figure 4:**
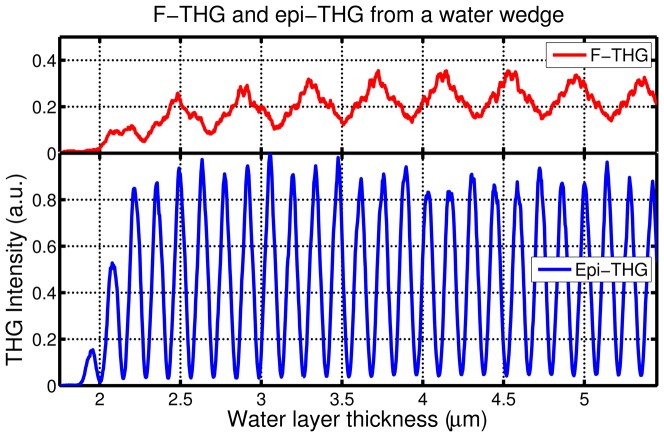
Epi-THG and F-THG from a water wedge. Epi-THG and F-THG from a water wedge between a microscope slide and a coverslip as a function of the water layer thickness. The relative detection efficiencies for epi-THG and F-THG were accounted for for direct comparison. The pixel dwell time is 300 ms.

By slightly tilting the scanning plane with respect to the excitation direction (*i.e.*, by 0.13° in Fig. 3c), fringes can be introduced to the epi-THG intensity pattern which can be used to quantitatively reconstruct the height pattern. We extracted the phase shift between the fringe patterns at the two different heights present in the patterned sample by fitting the phase profile (as found using cosine fits of the fringe pattern at a specific height) along a height step with a sigmoid function. By multiplying the fractional phase shift by a full fringe amplitude of 190 nm (see Figure 4), we determined that the etched pattern depth is 49 ± 3 nm, in good agreement with the depth of 45 ± 5 nm as measured with a profilometer. While we used knowledge of the pattern structure to extract the step size from a single image, multi-step interferometric methods can be used without such prior knowledge to determine the height variations accuracy far below the diffraction limit [12]. Clearly, structures with height variations much smaller than 49 nm can be visualized with interferometric THG microscopy.

### 4. Wedge cuvette sample

Further measurements of I-THG were performed on a glass wedge cuvette sample filled with distrilled water to quantify the resolution achievable with I-THG, and to investigate the relation between the epi-THG and F-THG intensity modulation with monotonously increasing distance between the two interfaces (Fig. 1c). The epi-THG and F-THG intensity as a function of the water layer thickness are shown in Fig. 4. The epi-THG signal is approximately 2.5 times more intense than the F-THG signal, which is caused by destructive interference of the F-THG signal in the forward direction due to the Gouy phase shift in the focused fundamental beam. The epi-THG signal does not suffer from such destructive interference, since the F-THG signal from the first glass layer is reflected by the two (probe and sample) interfaces and does not experience the out-of-phase F-THG from the second coverslip. The epi-THG fringes have a greater extinction ratio than F-THG (16 vs. 2). This confirms that interfaces other than the two in the focal volume do not contribute significantly to the epi-THG signal. The fringe spacing for epi-THG is approximately three times shorter than the fringe spacing for F-THG (139.3 ± 0.1 nm vs. 411 ± 2 nm). This is because the epi-THG fringes are due to the Fabry-Pérot effect acting on the F-THG signal from the first glass layer, while the F-THG fringes are caused by the Fabry-Pérot effect on the excitation beam. The epi-THG fringe spacing in air is approximately 190 nm due to the difference between the refractive indexes of distilled water and air. The higher frequency epi-THG oscillations are also observed in the F-THG signal due to the modulation of reflected F-THG by the two interfaces acting as a Fabry-Pérot etalon. All experimental observations correspond well with our theory and numerical predictions that were published elsewhere [6].

Using the epi-THG measurements we were able to unambiguously distinguish between differences in wedge spacing of 5 nm using the 10% to 90% range of the fringe amplitude, providing additional proof that height variations of a surface can be determined with an accuracy of several nanometers. Since the interferometric epi-THG method relies on the interference between the forward-generated beam reflected from the two interfaces, the accuracy for this stationary sample is in principle limited by the detection noise [12].

Furthermore, the ambiguity inherent in these fringe measurements can be mitigated in future work by using two different excitation wavelengths simultaneously, and by using the combined information to resolve a greater range of distances unambiguously.

## Conclusion

We have shown a new nonlinear microscopy technique based on interferometric epi-THG (I-THG) which can be used to image distance variations between interfaces with an accuracy of several nanometers. The height determination accuracy is limited mostly by the signal-to-noise ratio and the mechanical or inherent stability of the layers. Similar accuracy in surface height topography measurements can be achieved with linear interferometric techniques, including quantitative phase imaging (QPI) [14]. However, the I-THG technique presented in this paper can be used in multicontrast imaging, which allows for simultaneous acquisition of the height variation topography and, *e.g.*, multi photon excited fluorescence or harmonic generation images. In addition, nonlinear optical microscopy methods offer inherent optical sectioning, and therefore enable visualization of thickness variations of layers between interfaces or multilayers buried in a three-dimensional structure. Biological examples of such structures include adherent cell membranes in tissue or multilayer structures in mitochondria and chloroplasts.
